# Liver Abscess due to *Fasciola hepatica*: A Case Report of the North of Iran

**DOI:** 10.1155/2022/4399061

**Published:** 2022-06-25

**Authors:** Somaieh Matin, Farahnaz Joukar, Sara Yeganeh, Arash Daryakar, Fariborz Mansour-Ghanaei

**Affiliations:** ^1^Gastrointestinal and Liver Diseases Research Center, Guilan University of Medical Sciences, Rasht, Iran; ^2^Department of Internal Medicine, Emam Khomeini Hospital, Ardabil University of Medical Sciences, Ardabil, Iran; ^3^Caspian Digestive Diseases Research Center, Guilan University of Medical Sciences, Rasht, Iran; ^4^GI Cancer Screening and Prevention Research Center, Guilan University of Medical Sciences, Rasht, Iran

## Abstract

One of the rare manifestations of fascioliasis is liver abscess. In this paper, we report the case of a 38-year-old woman with a liver abscess caused by *Fasciola hepatica* (*F. hepatica*). The patient was referred to the clinic with recurrent fever, right upper quadrant (RUQ) pain, and a large abscess in her liver. Despite the consumption of an antibiotic drug, she still had symptoms. The symptoms began to disappear upon starting the consumption of triclabendazole (TCBZ). Fascioliasis can manifest itself with unusual symptoms that provide no specific clue for its diagnosis. Therefore, it is important to consider *F. hepatica* in the differential diagnosis of liver abscess, especially in endemic regions.

## 1. Introduction

Fascioliasis is a zoonotic disease which is caused by the trematode *Fasciola hepatica* (*F. hepatica*) [1]. The formation of liver abscesses is one of the rarest events that may be because of secondary to cholangitis or hepatic inflammation. Differentiating between amebic abscesses from the pyogenic form is difficult, and the use of clinical and laboratory examination for differentiating the two is essential [2]. Its diagnosis can be verified via demonstrating live parasites or parasite eggs in bile secretions or stool [3]. Based on scientific reports, it is estimated that 2.4 to 17 million people in more than 51 countries all over the world have been afflicted with this disease [4]. Several cases of this disease have been reported with a wide geographical distribution [5]. It has been highly prevalent in the developing countries [6, 7]. The largest global outbreak of this disease occurred in 1988 in Guilan Province (north of Iran), Iran, and affected 1000 people [8]. In this paper, we introduce a patient with a large liver abscess caused by *F. hepatica*, which was successfully treated with the consumption of TCBZ.

## 2. Patient Description

The patient was a 38-year-old woman from Rasht (a city in northern Iran which is the capital of Guilan Province) with no history of underlying chronic disease. She was referred to the clinic complaining of a gradually increasing epigastric and right upper quadrant (RUQ) pain in the last two months. According to the patient's self-report, the pain was not associated with eating, was not positional, and did not spread to other organs of the body. The patient had no history of consuming herbal medications, eating nutritional supplements, smoking, or being in touch with pets. Also, she did not mention any history of urticaria, food allergies, or family history of liver disease. She had not travelled anywhere during the past months. In addition to suffering from abdominal pain, the patient also complained of fever and chills, non-productive coughs, anorexia, and nausea after eating food. She usually had irregular and intermittent fever for 2 months in the afternoon and grappled with it until late at night before treatment. She usually started having fever in the afternoon and grappled with it until late at night. Then, her body temperature would get back to normal without consuming any specific medication. The fever cycles occurred every 1–3 days and the patient had normal body temperature between the cycles. During the 2-month period before referral to the clinic, she had experienced fever cycles with this pattern for about 15 days (5 to 7 cycles). Besides having fever, the patient also complained of occasional joint pain in those fever cycles. The patient had lost 7 kilos of her weight during the past 2 months. The symptoms of the disease were deteriorating. Severity of abdominal pain was the main complaint during the past 2 months. Based on preliminary examination, the patient was alert and oriented. As for vital signs, the patient's blood pressure was 115/75 mm·Hg, her respiratory rate was 15 breaths per minute, her heart rate was 86 beats per minute, and her body temperature was 37.5°C. Except for diffuse wheezing in the lungs and tenderness in RUQ, physical examination did not indicate any other pathological finding.

**Figure 1:**
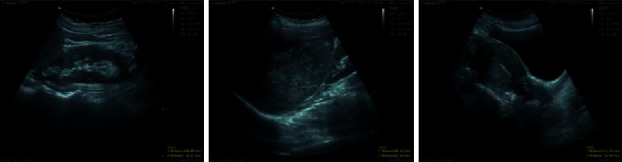
Patient's hepatobiliary ultrasound.

Ultrasound images of liver and bile ducts revealed the existence of a heterogeneous hypoechoic mass with lobulated border containing cystic lesions with the diameter of 90 *∗* 62 mm in segments 6 and 8 of the liver (Figure 1). With the possible diagnosis of liver abscess, an antibiotic drug including ceftriaxone 2 gr intravenously (IV) once daily plus metronidazole 1.5 gr IV daily was prescribed for the patient's treatment. The patient's body, however, did not respond to this treatment. Her pain exacerbated and she also started to have coughs. Therefore, she was referred to a specialized gastroenterology and liver clinic for further examinations.

**Figure 2:**
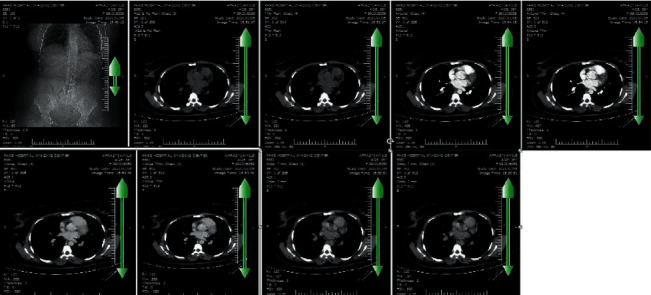
Multiple images of the patient's CT scan.

The abdominal computed tomography (CT) scan report was as follows. There is a large, ill-defined multiloculated, predominantly hypodense lesion (measuring 90 ^*∗*^ 80 mm) with multiple internal central cystic areas in segments 5 and 6 of the liver. The lesion shows hypodense than normal parenchyma in arterial to equilibrium phase at post-contrast study without hemangioma imaging feature. Several lymph nodes in porta hepatis, paracaval, and para-aortic regions (maximum SAD = 11 mm in left para-aortic region) are seen. There is also a small poorly defined hypodense lesion (measuring 2 ^*∗*^ 3 mm) in segment 6. Post-contrast images show centripetal enhancement at porto-venous phase (Figure 2).

**Figure 3:**
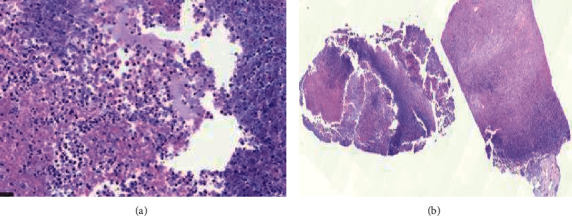
Histopathological images of liver biopsy. (a) Abscess wall composed of infiltration of neutrophils, some lymphocytes, and macrophages. (b) Liver abscess formation, low power view.

Considering the prevalence of COVID-19, the patient was tested for this disease with high-resolution computed tomography (HRCT) and polymerase chain reaction (PCR) tests, the results of which were negative. Similarly, the results of endoscopy and colonoscopy were negative rejecting the existence of malignancies (Figure 3). The complete blood count was performed, and the results indicated 32% of eosinophils in the blood, based on which the patient was suspected of parasitic infection. The results of serologic tests for amoebae and *Echinococcus granulosus* were negative. However, the result of the serologic test for *F. hepatica*, which was performed using Falcon assay screening test (FAST-ELISA), was positive (Table 1). Following this observation, antiparasitic treatment was started with TCBZ at the dosage of 10 mg/kg every 12 hours for two days. For definitive diagnosis, the patient underwent ultrasound-guided biopsy. Necrosis pathology indicated acute inflammation and abscess formation in accordance with the processes observed in various types of infections including parasitic infections without any malignancies.

## 3. Discussion

Liver abscess is a rare manifestation of fascioliasis. So far, 36 cases of acute liver abscess caused by *F. hepatica* have been reported in 13 case report studies all over the world [9–21]. Most of them have reported RUQ pain, fever, eosinophilia, and anemia in the afflicted patients, which are all consistent with our findings.

Prolonged fever [15, 22] and recurrent fever are among the rare symptoms of this disease [23]. The case under investigation in the current study had been referred to the clinic with the preliminary symptom of recurrent fever. In another study, which was conducted in a southern city of Iran, the presented case was reported to have prolonged fever [24].

In the studies conducted by Olivier et al. in France and Vatan et al. in Turkey, RUQ pain was reported in patients under investigation [25, 26]. Their findings in this regard are consistent with the findings of the current study.

In Egypt and Turkey, an increase in alanine aminotransferase (ALT) and aspartate aminotransferase (AST) was reported [27]. Also, in the study conducted by Ghaderi et al. in Iran, alkaline phosphatase (ALP) and AST were observed to increase [24]. In the current study, however, only ALT was observed to increase.

The increase of C-reactive protein (CRP) in the present study is consistent with the findings reported by Vatan et al., Olivier et al., and Ghaderi et al. [24–26]. The patient was also observed to have anemia, which is again consistent with the findings reported by Ghaderi et al. [24]. In addition, an increase was observed in erythrocyte sedimentation rate (ESR) in this study, which was in line with the findings reported by Elshazly et al. [27]. Moreover, the reduction of the patient's blood pressure in the current study was consistent with the findings reported by Vatan et al. [26].

In our study, the test results were indicative of eosinophilia in the patient's blood. In the study conducted by Elshazly et al. in Egypt, eosinophilia was observed in 100% of the patients [27]. In Mansouri et al.'s study, which was conducted in Iran, eosinophilia was observed approximately 2 months after disease manifestation and severe respiratory problems [28]. Eosinophilia is another common manifestation of fascioliasis along with the other clinical symptoms. However, in rare cases, this disease can occur without eosinophilia [26, 27, 29].

*F. hepatica* is usually seen in the form of hypoechoic lesion in ultrasound images and hypodense lesion in CT scan images [30]. In this study, ultrasound images of liver and bile ducts indicated a heterogeneous hypoechoic mass with lobulated border containing cystic lesions with a diameter of 90 *∗* 62 mm. No specific CT scan finding was observed for *F. hepatica* in this study that matched with other studies [9–19]. In the obtained images, the hypodense lesion was similar in shape to amoebic or pyogenic liver abscesses. Hepatic portal vein thrombosis with liver abscess was observed in only one of the studies [17]. In the study conducted by Olivier et al. in France, CT scan and MRI images indicated several hypodense lesions together with abscess in the right lobe of the liver. The largest hypodense lesion was reported to be 40 mm [25]. In addition to showing hypodense nodules and liver abscesses, CT scan images can also depict the movement of *F. hepatica* in the liver tissue [25, 31, 32]. The CT scan image of the case under investigation in the present study demonstrated a large hypodense mass with the size of 90 *∗* 82 mm together with several cysts and lymph nodes in porta hepatis, paracaval, and para-aortic areas.

In this study, after detecting liver abscess and starting treatment with antibiotics and TCBZ, the patient's symptoms began to improve. In the study conducted by Ghaderi et al., the patient was referred to the clinic with severe symptoms and was diagnosed to have liver abscess. Then, treatment began with an antibiotic drug. It was expected that at the end of the treatment course, the abscess would disappear completely. However, neglecting *F. hepatica* led to the recurrence of the disease. Eventually, after the positive result of the serologic test for *F. hepatica*, the patient's treatment began with TCBZ at the dosage of 10 mg/kg, as the result of which the liver abscess disappeared and the fever was reduced. The CT scan image taken 5 months later showed that all lesions in the liver had disappeared [24]. TCBZ has widely been used for the treatment of fascioliasis and has proved to be effective [33]. This drug can be tolerated easily and has no major side effect [34], which was the case in our patient, too.

After 3 months of follow-up, the patient fully recovered and the imaging data demonstrated a significant reduction in the size of the abscess.

In our study, the differential diagnoses of liver masses including amoebic abscesses, tuberculosis, malignancies, and liver metastasis were all propounded and assessed. However, they were all rejected after supplementary investigations. Finally, in view of the high percentage of eosinophils, the positive result of the serologic test for *F. hepatica*, the patient's positive response to treatment with TCBZ, and the relatively high prevalence of fascioliasis in the patient's living environment, the final diagnosis was confirmed to be fascioliasis.

## 4. Conclusion

In this case report, we emphasized that fascioliasis might manifest itself through unusual symptoms with no specific clue as to its underlying cause. Therefore, it is suggested that *F*. *hepatica* be considered in the differential diagnoses of liver abscesses, especially in endemic regions. By doing so, the problem can be diagnosed earlier, therapeutic interventions can begin sooner, and invasive diagnostic tests can be avoided.

## Figures and Tables

**Table 1:** Lab findings of the patient's tests.

Test	Results	RV
WBC	8.8 × 10^3^/mm^3^	4–11 × 10^3^/mm^3^
Neut	34	(%)
LYM	29	(%)
EOS	32	(%)
MONO	4	(%)
Hb	11.7	14–18 g/dL
Hct	36.5	40–57%
MCV	82.3	80–100 femtoliter
PLT	278 × 10^3^	150–450 × 10^3^ cumm
AST	34	(5–40 IU/L)
ALT	50	(5–40 IU/L)
ALP	274	80–306 IU/L
T Bil	0.9	0.1–1.2 mg/dl
PT	12	11–13.5 sec
INR	1.1	
PTT	35	25–40 s
ESR	85	0–25 mm/hr
CRP	143	<6 mg/dl
Alb	4.5	3–5 g/dl
AFP	1.05	<9 mg/dl
CA19-9	41	<40 U/ml
CEA	1.6	<4.5 ng/ml
S Ab	8.8	<9 unit
F Ab	3.8	<0.9 unit
H IgG	Negative	
Stool OB	Normal	

RV: reference value; WBC: white blood cells; Neut: neutrophil; LYM: lymphocyte; EOS: eosinophil; MONO: monocyte; Hb: hemoglobin; Hct: hematocrit; MCV: mean corpuscular volume; PLT: platelet; AST: aspartate aminotransferase; ALT: alanine aminotransferase; ALP: alkaline phosphatase; T Bil: total bilirubin; PT: prothrombin time; INR: international normalized ratio; PTT: partial thromboplastin time; ESR: erythrocyte sedimentation rate; CRP: C-reactive protein; Alb: albumin; AFP: alpha fetoprotein; CA19-9: carbohydrate antigen: CEA: carcinoembryonic antigen; S Ab: strongyloides Ab; F Ab: fasciola Ab; H IgG: hydatid IgG; Stool OB: stool occult blood.

## Data Availability

The data used to support the study are available from the corresponding author upon request.
